# Vitamin D status among adults in the Aegean region of Turkey

**DOI:** 10.1186/1471-2458-10-782

**Published:** 2010-12-23

**Authors:** Zeliha Hekimsoy, Gönül Dinç, Sabriye Kafesçiler, Ece Onur, Yesim Güvenç, Tümer Pala, Feyzullah Güçlü, Bilgin Özmen

**Affiliations:** 1 Celal Bayar University Medical Faculty, Department of Internal Medicine, Division of Endocrinology and Metabolism, Manisa, Turkey; 2 Celal Bayar University Medical Faculty, Department of Public Health, Manisa, Turkey; 3 Celal Bayar University Medical Faculty, Department of Clinical Biochemistry, Manisa, Turkey; 4 Celal Bayar University, Vocational School of Health Services, Department of Biochemistry, Manisa, Turkey; 5 Ministry of Health, Directorate General of Health for Border and Coastal Areas, Izmir, Turkey

## Abstract

**Background:**

Vitamin D is a lipid-soluble hormone found in certain foods and synthesized from precursors in the skin when exposed to ultraviolet light. Vitamin D plays a critical role in bone metabolism and many cellular and immunological processes and low levels have been associated with several chronic and infectious diseases. Vitamin D status is assessed by measuring the concentration of serum 25-hydroxyvitamin D [25(OH)D]. Vitamin D deficiency is reported to be common worldwide, but little has been reported about the vitamin D status of adults in Turkey. In this cross-sectional study, we determined the prevalence of 25(OH)D deficiency in adults residing in a city in the Aegean region of Turkey.

**Methods:**

A survey was conducted on a representative sample of adults over 20 years old in a non-coastal city at the end of the winter season. Of the 209 households selected by random sampling, 8.6% (n = 18) were unoccupied and 21.5% (n = 45) refused to participate. Blood samples were taken and questions about medical history, vitamin supplementation, sunlight exposure, and dietary calcium and vitamin D intake were asked in face-to-face interviews of 391 adults living in the remaining households.

**Results:**

The mean serum 25(OH)D concentration was 16.9±13.09 ng/mL, with 74.9% of the subjects having 25(OH)D deficiency (<20 ng/mL), 13.8% having insufficiency (20-29.99 ng/mL), and 11.3% of the subjects having sufficient 25(OH)D (≥30 ng/mL) levels. 25(OH)D deficiency was more common among females (78.7%) than males (66.4%, p < 0.05).

**Conclusion:**

Adults living in an urban, non-coastal setting in Turkey have a high prevalence of vitamin D deficiency.

## Background

Vitamin D is a lipid-soluble hormone found in certain foods and synthesized from precursors in the skin when exposed to ultraviolet light [1-10]. Vitamin D plays a critical role in bone metabolism and many cellular and immunological processes; and low levels have been associated with several chronic and infectious diseases including diabetes [11-13], cardiovascular diseases [6], cancer [14-17], multiple sclerosis [18,19], osteoporosis [9], autoimmune diseases [20], and microbial infections [21]. Vitamin D deficiency is generally due to a lack of adequate sunlight exposure or excessive use of sunscreen, and/or inadequate nutritional vitamin D intake [10]. Tropospheric ozone, a common pollutant in central urban areas, is also associated with reduced synthesis of vitamin D in the skin and resulting vitamin D deficiency [22]. Several other factors such as skin pigmentation, age, weight, malabsorption disorders and disorders that affect vitamin D metabolism can also affect vitamin D concentrations [1-10]. Plasma (or serum) 25(OH)D is currently the best indicator for vitamin D status (1). This is a measure of the circulating concentration of serum 25(OH)D, which reflects vitamin D from all sources: cutaneous synthesis, diet and supplements [1,9].

Although there is no consensus on optimal serum levels of 25(OH)D, vitamin D deficiency is defined by most experts as a level less than 20 ng/mL [1]. Vitamin D deficiency is a highly prevalent condition, present in approximately 30% to 50% of the general population [6]. This worldwide pandemic remains generally unrecognised and untreated. To date, little has been reported about the vitamin D status of adults in Turkey [23-26]. In this cross-sectional study, we determined the prevalence of vitamin D deficiency in adults residing in a city in the Aegean region of Turkey.

## Methods

### Setting

This cross-sectional study was conducted in the city of Manisa (38.36º N latitude), a non-coastal city in the Aegean region of Turkey with a population of 303,155 [27]. The population was 55% urban, 30% semiurban and 15% rural. Clothing covering the whole body (face included), while common in some countries (eg. burqa), is very rarely worn in western Turkey. The study protocol was approved by the Institute of Health and Human Development Ethical Issues on Clinical Trials Review Board of Celal Bayar University.

In 2007, when the data was collected, the winter was drier and sunnier than normal. The average duration of sunlight in this region is 0 to 9 hours per day in winter and 0.30 to 11 hours per day in summer. The minimum and maximum temperatures of this region range from -2.4° to 19.7°C in winter and 15° to 45°C in summer.

### Study sample

The study sample was selected from among a population of 205,940 persons over 20 years old who granted consent to participate in the study. Exclusion criteria consisted of a history of liver disease, renal failure, or cancer, or calcium or vitamin D supplement use or the presence of a parathyroid problem (n = 0). Using an estimated prevalence of vitamin D deficiency in the population of 30% (p), and accepting a 5% type 1 error, the sample size needed to reach a power of 80% with a precision of 4% (s) in reporting the prevalence of vitamin D deficiency was calculated to be 504 individuals.

$$\text{n}=\frac{\text{p}\left(1-\text{p}\right){\text{t}}_{0.05}}{{\text{s}}^{2}}=\frac{\left(0.30\right)\times \left(0.70\right)\times {\left(1.96\right)}^{2}}{\left(0.04\right)\times \left(0.04\right)}=504\;\text{individuals}$$

During the study period, the primary health care system of Turkey covered the whole population, and was organized through health center districts. Sample groups were selected randomly from records of households of three different primary health centers (urban/semiurban/rural) using systematic sampling methods. The number of households for each neighborhood were determined according to neighborhood's proportional population in the city and the neighborhood's mean household size over 20 years (2.4 persons per household, according to official records). Overall, 209 households were randomly selected, and adults convenient for participation in the study were asked to participate, ie. some households had more than one adult participate in the study.

### Data collection

The data were collected in the winter, in February of 2007. A structured questionnaire (previously tested for comprehension on 10 women and 10 men who were in the hospital for ambulatory care) was answered during face-to-face interviews with the study population in their homes. Demographic data was collected, as well as information concerning the family's socio-economical level, age, gender, occupation, family income, highest educational level, chronic diseases, medications, dietary intake of calcium and vitamin D, and habits and clothing which preclude exposure to sunlight (see below). Weight and height were directly measured, from which body mass index (BMI, kg/m^2^) was calculated.

Ten mL of blood was collected from a peripheral vein, quickly placed on ice, and transported to the laboratory where it was centrifuged at 4000 rpm for four minutes, then serum and plasma were stored at -70°C until analysis. Serum was tested for the following biochemical parameters: 25(OH)D, parathyroid hormone (PTH), calcium, phosphorus, alkaline phosphatase, creatinine and albumin.

### Biochemical parameters

PTH levels were measured by chemiluminescence method on an IMMULITE 2000^® ^analyzer (Diagnostic Products Corporation, Los Angeles, USA). 25(OH)D levels were measured by HPLC (Thermo-Finnigan, Waltham, USA) using Vitamin D3 ClinRep HPLC kits (RECIPE Chemicals+Instruments GmbH, Munich, Germany). The method involves a 50 μL injection volume of the sample in a mobile phase which has a flow rate of 1.0 mL/minute. Measurements are calculated by using internal standard-methods via peak areas. For the determination of the within-run precision, the samples were measured in duplicate. The average coefficient of variation was 4.8%. Since the measurements were done on the same day, intra-assay precision was not calculated. However, the manufacturer claims a 2.3-4.9% coefficient of variation for a concentration of 21.1 μg/L.

### Terms and definitions

#### Assessment of exposure to sunlight

We did not directly measure exposure to sunlight. On the questionnaire, the interviewer asked about being inside between 10 a.m. and 4 p.m, wearing of a hat or long-sleeve shirts/skirts when outside, and use of sunscreen and/or an umbrella when outside. A Likert scale was used for each question: always (1 point), usually (2 points), sometimes (3 points), and never (4 points). Adding up the points for these questions gave us a 'sun exposure index' ranging from a low of 4 points to a maximum of 16 points. Sun exposure was considered mild (< 8 points), moderate (8-11 points) or high (12-16 points). Regular wearing of clothing limiting exposure to sunlight was also asked, as a yes/no question. 'Yes' was recorded if usually the person wore clothing which allowed for only the face and hands to be exposed to the sun. If otherwise, 'no' was recorded.

#### Vitamin D status

Vitamin D nutritional status was assessed as deficient if 25(OH)D levels were <20 ng/mL, insufficient if between 20 and 30 ng/mL, and sufficient if ≥30 ng/mL [1].

#### Other

Mean daily calcium intake was determined according to the daily diet history. Present medical problems and medications were elicited by the interviewer.

### Statistical analyses

All statistical analyses were performed using SPSS for Windows^® ^11.0 (SPSS, Inc. Chicago, USA). Chi-square test and Student's t test were used for bivariate analysis. Two logistic regression models were developed to examine the associations between vitamin D levels and risk factors. The adjusted odds ratios and their corresponding 95% confidence intervals (CIs) were calculated. Sex (male/female), age (<50 or ≥50 years), clothing limiting exposure to sunlight (Yes/No), and area of residence (urban/semiurban/rural) were included as potential confounders in the multivariate analysis. Independent variables (age, sex, wearing of clothing which limits exposure to sunlight, and household location) were compared by logistic regression in patients with various levels of vitamin D, using two different models: vitamin D deficiency or insufficiency vs sufficiency (Model I) and vitamin D deficiency vs. sufficiency (Model II). P values less than 0.05 were regarded as statistically significant.

## Results

Of the 209 randomly selected households, 8.6% (n = 18) were unoccupied and 21.5% (n = 45) of the households refused an interview. Thus the overall rate of participation was 70% (64% in urban, 89% in semiurban, and 53% in rural areas). Thus, households from the semiurban neighborhoods were overrepresented in our population sample.

We enrolled 391 subjects in the study (mean age 45.11 ± 17.28 years): 30.4% male (n = 119, mean age 45.05 ± 17.47 years), 69.6% female (n = 272, mean age 45.13 ± 17.23 years), and 62.7% were less than 50 years old. Of the participants, 39.6% were from urban, 49.4% from semiurban, and 11.0% from rural areas (Table 1). No patient was taking calcium or vitamin D as a supplement. None had a parathyroid problem, chronic kidney disease, liver disease or other medical conditions which might affect vitamin D metabolism.

**Table 1:** Demographic characteristics, sunlight exposure, reported calcium intake, and mean 25(OH)D levels of the participants.

	Male (n = 119)	Female (n = 272)	Total (n = 391)	P
**Total**	30.4%	69.6%	100.0%	

**Age**				*p = 0.6
<50 years	62.4%	62.9%	62.7%	
≥50 years	37.6%	37.1%	37.3%	

**Area of residence**				*p = 0.1
Urban	46.2%	36.8%	39.6%	
Semi-urban	45.4%	51.1%	49.4%	
Rural	8.4%	12.1%	11.0%	

**Wearing clothes which restrict exposure to sunlight**				*p = 0.001
Yes	55.8%	72.6%	67.6%	
No	44.2%	27.4%	32.4%	

**Sunlight exposure index**				*p = 0.0009
<8 points	0.9%	6.1%	4.5%	
8-11 points	30.4%	49.6%	43.8%	
≥12 points	68.7%	44.3%	51.7%	

**Daily calcium intake**				*p = 0.8
500 mg/day	19.3%	20.2%	19.9%	
<500 mg/day	80.7%	79.8%	80.1%	

**Mean ± SD 25(OH)D level (ng/mL)**	20.70 ± 15.50	15.25 ± 11.53	16.91 ± 13.09	**p = 0.001

*Chi-square testing

**Student's t test

Overall, 67.6% of the respondents regularly wore clothing that prevented exposure to sunlight (72.6% in women, 55.8% in men, p < 0.001, Table 1). Regarding the sun exposure index, 4.5% had mild, 43.8% had medium, and 51.7% had high exposure to sunlight. Women had less exposure to sunlight than men (p < 0.001, Table 1). Calcium intake was low in the respondents (Table 1).

The overall mean 25(OH)D level was 16.91 ± 13.09 ng/mL; levels were lower in women than in men (15.25 ± 11.53 ng/mL vs 20.70 ± 15.50 ng/mL, respectively, p = 0.001, Student's t test, Table 1). 25(OH)D status was not significantly different when analyzed according to age, BMI, and area of residence (Table 2). Overall, 74.9% had vitamin D deficiency, 13.8% had vitamin D insufficiency, and only 11.3% had sufficient levels of vitamin D. Vitamin D deficiency was significantly more common among women (78.7%) than men (66.4%, p < 0.01). 25(OH)D levels were not significantly different in persons according to age, BMI, or household location.

**Table 2:** Vitamin D status and age, gender, body mass index (BMI) and area of residence.

		25(OH)D status		
		
	Deficient (<20 ng/mL)	Insufficient (20-29.99 ng/mL)	Sufficient (≥30 ng/mL)	p*
**Total (n = 391)**	74.9%	13.8%	11.3%	

**Age (years) (n)**				p = 0.6
<50 (n = 247)	75.7%	14.2%	10.1%	
≥50 (n = 144)	73.6%	13.2%	13.2%	

**Gender**				**p = 0.025**
Male (n = 119)	66.4%	20.2%	13.4%	
Female (n = 272)	78.7%	11.0%	10.3%	

**BMI (kg/m^2^)**				p = 0.9
<25 (n = 151)	74.0%	14.0%	12.0%	
25-29.99 (n = 152)	74.3%	15.8%	9.9%	
≥30 (n = 88)	69.0%	19.0%	12.1%	

**Area of residence**				p = 0.07
Semiurban (n = 193)	70.5%	14.5%	15.0%	
Urban (n = 155)	76.8%	14.8%	8.4%	
Rural (n = 43)	88.4%	7.0%	4.7%	

*Chi-square test

25(OH)D levels according to style of dress and sunlight exposure are given in Table 3. Those wearing clothing which restricted exposure to sunlight were more likely to have vitamin D deficiency (77.2%) than those who wore clothing which allowed more sunlight exposure (71.3%). In the same way, those wearing clothing which restricted exposure to sunlight were more likely to have vitamin D insufficiency (14.6%) than those who wore clothing which allowed more sunlight exposure (11.5%, p = 0.032, Table 3). However, sunlight exposure, as measured by our scale, and daily calcium intake, were not significantly associated with 25(OH)D status.

**Table 3:** Vitamin D status and clothing, sunlight exposure and calcium levels

		25(OH)D status		
		
	Deficient (<20 ng/mL)	Insufficient (20-29.99 ng/mL)	Sufficient (≥30 ng/mL)	P*
**Wearing clothes which restrict exposure to sunlight**				**p = 0.032**
Yes	77.2%	14.6%	8.3%	
No	71.3%	11.5%	17.2%	

**Sunlight exposure**				p = 0.570
<8 points	82.4%	11.8%	5.9%	
8-11 points	78.2%	10.9%	10.9%	
≥12 points	71.8%	15.9%	12.3%	

**Daily calcium intake**				p = 0.880
500 mg/day	76.0%	14.7%	9.3%	
<500 mg/day	74.8%	13.9%	11.3%	

*Chi-square test

Results of the logistic regression models are presented in Table 4. Those who regularly wore clothing which restricted skin exposure to sunlight were more likely to have vitamin D deficiency or insufficiency (Table 4). The odds of being vitamin D deficient was over two times as high for those living in urban areas compared to semiurban areas (Table 4).

**Table 4:** Logistic regression models regarding 25(OH)D levels, with the following independent variables: sex (male/female), age (<50/≥50 years), wearing of clothing which limits sunlight exposure (yes/no), and household location (urban/semiurban/rural).

	Model I: (vitamin D deficiency or insufficiency vs sufficiency)		Model II: (vitamin D deficiency vs sufficiency)	
	**Odds ratio (95%CI)**	**p value**	**Odds ratio (95% CI)**	**p value**

**Age (years)**				
<50 (reference)	1		1	
≥50	0.55 (0.28-1.09)	0.090	0.56 (0.28-1.13)	0.107

**Gender**				
Male (reference)	1		1	
Female	1.28 (0.63-2.57)	0.484	1.47 (0.72-3.00)	0.282

**Wearing clothes which restrict exposure to sunlight**				
Yes	2.90 (1.45-5.81)	**0.003**	2.63 (1.31-5.28)	**0.006**
No (reference)	1		1	

**Area of residence**				
Semiurban (reference)	1		1	
Urban	2.47 (1.17-5.21)	**0.017**	2.42 (1.14-5.12)	**0.020**
Rural	4.13 (0.91-18.58)	0.064	4.19 (0.93-18.91)	0.062

r^2^	0.091		0.098	

## Discussion

Vitamin D deficiency is being increasingly recognised worldwide [1,9]. Studies among those living in Turkey have been few, and most have been performed among women, those in nursing homes, and the elderly [23-26]. To date, no study has examined vitamin D status in a population based sample in Turkey. This is the first such study, and found quite a high prevalence of vitamin D deficiency (74.9%) and insufficiency (13.8%), quite a high proportion for an area of the country which gets lots of sunlight.

Other reports in the literature have found vitamin D deficiency (as defined by a 25(OH)D level of less than <20 ng/mL) in 40-100% of those tested, with proportions varying according to geographic area, latitude and specifics of the patient population [1,28-30]. The Third National Health and Nutrition Examination Survey (NHANES III) reported the prevalence of vitamin D deficiency in the U.S.A. to be between 25% and 57% of adults [28]. Van der Wielen et al. [29] measured wintertime serum 25-hydroxyvitamin D concentrations in 824 elderly people from 11 European countries and found vitamin D levels lower than 20 ng/mL in 36% of men and 47% of women. Surprisingly, vitamin D deficiency was much more common in people living in sunny countries such as Italy, Spain and Greece than among those living in Scandinavian countries where sunlight exposure is less. Higher fish consumption and vitamin D fortification of food could explain this difference [29].

The mean 25(OH)D level was low (16.91 ± 13.09 ng/mL) in our study population. As in other studies [23,26,30-32], vitamin D deficiency was more common in women than in men (78.7% vs 66.4%) and mean 25(OH)D levels in women were significantly lower than in men. Kull et al. [33] reported mean wintertime 25(OH)D levels to be 17.2 ± 5.9 ng/mL in the general population of Estonia (latitude 59°C). The prevalence of vitamin D deficiency in their study was 8% and insufficiency 73%, which are slightly lower than in our population, although Estonia is located much farther north and receives less sunlight than the Aegean region of Turkey.

Vitamin D is recognized as the sunshine vitamin. Skin synthesis of vitamin D is the major source of this vitamin. More than 90% of the vitamin D requirement for most people comes from casual exposure to sunlight. Natural dietary sources of vitamin D are limited, unless fortification or supplementation practices are adopted [34,35]. Regular sunlight exposure acts as effective prophylactic to prevent vitamin D deficiency. But even in Middle Eastern populations, who live in sunny climates, vitamin D deficiency is present in 50-97% of those tested [32,36,37], most probably due to wearing of clothes which leave little skin exposed to sunlight. Our population also had a high percentage (67.6%) who wore clothing which restricted sunlight exposure; this proportion was higher among women than among men. Overall sun exposure was also low in our population, with only 51.7% reporting a high degree of sun exposure, men having more sunlight exposure than women. Although our method for measuring skin exposure to sunlight was not objective, as in cm^2 ^exposed, we feel that classification of the clothing habits of our population was a reasonable proxy for this variable.

As expected, we found a higher prevalence of vitamin D deficiency and insufficiency among those with less exposure to sunlight due to their clothing habits. However, the degree of overall sun exposure was not predictive of vitamin D status. We can not explain exactly why urban participants were more than two times likely to have vitamin D deficiency or insufficiency than those residing in semiurban areas. Tropospheric ozone is a common pollutant in central urban areas and may reduce synthesis of vitamin D in the skin; this may then result in vitamin D deficiency [22]. However, in our population, the differences in prevalence of vitamin D deficiency are likely due to traditional dress in rural areas allowing less sunlight exposure to the skin.

This first randomly selected population-based survey indicates that vitamin D deficiency is very prevalent in the Aegean region of Turkey. Our results reflect those of the population living in one city and its surroundings; these results may differ markedly from those in other regions due to differences in climate, dress, nutrition, and sociodemographics. For this reason, a population-based study of vitamin D status should be performed throughout Turkey as a whole.

## Limitations

Several limitations were present in our study. First, only 70% of our randomly chosen households were available and agreed to be interviewed. Those refusing interviews, or those not at home, might have different vitamin D status than those at home, or those consenting to interviews. A greater number of our participants than planned were from the semiurban area, thus our results are reflective of this bias. Another issue facing our researchers was the presence of women, not men, in the homes during working hours, leading to 69.6% of our participants being female. Because women were overrepresented in our population sample, the overall mean 25(OH)D level would probably have been slightly higher if more men had been sampled. The fact remains though that only about 10% of men and women in the Aegean region of Turkey have an adequate vitamin D status.

Another limitation is one present in many surveys, that of taking a dietary history by survey instead of by direct observation or daily diary. Participants may not have given accurate information regarding their calcium intake.

Because this was a cross-sectional study, the reason for low vitamin D levels cannot be proved, only an association with lifestyle and dietary factors can be shown. A carefully performed longitudinal study would be required to examine causation of vitamin D deficiency.

## Conclusion

This population-based survey of households in the Aegean region of Turkey indicates that vitamin D deficiency is very prevalent. A nationwide population-based study is needed to determine the vitamin D status of persons in different locales throughout Turkey. Based on responses of our participants, lifestyle changes to increase sunlight exposure would increase vitamin D levels. A broad-based effort to prevent vitamin D deficiency in Turkey should be undertaken.

## Competing interests

The authors declare that they have no competing interests.

## Authors' contributions

All authors contributed to the concept, design, analysis, interpretation of data, and preparation of the manuscript. All authors read and approved the final manuscript.

## Pre-publication history

The pre-publication history for this paper can be accessed here:

http://www.biomedcentral.com/1471-2458/10/782/prepub
